# Advancing Genomic Surveillance of Human Parainfluenza Viruses in Côte d'Ivoire Using Twist Hybrid‐Capture Sequencing

**DOI:** 10.1111/irv.70335

**Published:** 2026-10-14

**Authors:** Olivia Cariolh Koumba‐Koumba, Yakoura Karidja Ouattara, Herve A. Kadjo, Mouhamed Kane, Maxime Diané Kouao, Helene Kouassi, Ange‐Michele Mbra, Alida Mouliom, Ndeye Awa Ndiaye, Ndongo Dia, Daouda Coulibaly, Edgard Adjogoua, Therese K. Agbessi, Moussa Moise Diagne

**Affiliations:** ^1^ Department of Epidemic Viruses Institut Pasteur de Côte d'Ivoire Abidjan Côte d'Ivoire; ^2^ UFR of Medical Sciences Université Félix Houphouët‐Boigny Abidjan Côte d'Ivoire; ^3^ Virology Department Institut Pasteur de Dakar Dakar Senegal; ^4^ Epidemiological Surveillance Service National Institute of Public Hygiene Abidjan Côte d'Ivoire; ^5^ Department of Pharmaceutical and Biological Sciences Université Félix Houphouët‐Boigny Abidjan Côte d'Ivoire

**Keywords:** Côte d'Ivoire, genomic surveillance, HPIV, human parainfluenza virus, hybrid‐capture sequencing, phylogenetics, respiratory viruses, West Africa

## Abstract

**Background:**

Human parainfluenza viruses (HPIV1–3) are major causes of acute respiratory infections, yet their genomic epidemiology remains poorly characterized in sub‐Saharan Africa. We evaluated hybrid‐capture sequencing for HPIV genomic surveillance in Côte d'Ivoire using respiratory samples collected through the national sentinel surveillance network from January 2022 to December 2024.

**Methods:**

Among 268 RT‐qPCR‐positive samples, 49 were selected for sequencing using the Twist Respiratory Virus Research hybrid‐capture panel on the Illumina iSeq 100 platform. Genome assembly and phylogenetic analyses were performed using CZ‐ID and IQ‐TREE.

**Results:**

Hybrid‐capture sequencing recovered 29 near‐complete genomes: 9 HPIV1, 10 HPIV2, and 10 HPIV3. Genome recovery was strongly dependent on viral load, with a marked negative correlation between cycle threshold (Ct) value and genome coverage (*r* = −0.91) and optimal performance at Ct ≤ 30. Viral co‐detections were identified in 25 sequenced samples; adenovirus type B was the most frequent co‐detected pathogen, while HPIV3 showed the broadest co‐detection profile. Phylogenetic analysis revealed limited diversity for HPIV1, greater diversity for HPIV2, and pronounced heterogeneity for HPIV3, including co‐circulation of divergent lineages.

**Conclusions:**

Hybrid‐capture sequencing provides a practical approach for HPIV genomic surveillance in routine settings and offers detailed insight into HPIV circulation in West Africa, supporting lineage tracking and the design of future genomic surveillance strategies.

## 1 Introduction

Human parainfluenza viruses (HPIV1–4), members of the family *Paramyxoviridae*, are major causes of acute respiratory infections worldwide, particularly among young children, older adults, and immunocompromised individuals. Globally, HPIVs account for a substantial proportion of lower respiratory tract infections in children and contribute significantly to morbidity and healthcare burden, especially in low‐resource settings [1, 2, 3]. HPIV infections do not induce long‐lasting protective immunity, thereby predisposing individuals to recurrent infections throughout life. Moreover, no licensed vaccine or specific antiviral therapy is currently available, underscoring the importance of surveillance and a better understanding of their evolutionary dynamics [4]. Genomic surveillance has become an essential tool for characterizing the genetic diversity of respiratory viruses, tracking lineage introductions and circulation, and supporting epidemiological interpretation of outbreaks and seasonal trends. However, unlike other major respiratory pathogens such as influenza virus or SARS‐CoV‐2, HPIVs remain relatively underrepresented in genomic databases, with historically limited numbers of complete genomes publicly available. This scarcity hampers high‐resolution phylogenetic analyses and long‐term evolutionary studies [4].

In sub‐Saharan Africa, this gap is even more pronounced. Virological surveillance systems largely rely on targeted diagnostic approaches, while the generation of genomic data remains constrained by technological, logistical, and human resource limitations [5]. In West and Central Africa, several studies have documented the regular circulation of HPIV among children presenting with acute respiratory infections, confirming their contribution to the regional respiratory disease burden [6, 7]. However, these investigations were primarily based on targeted molecular diagnostics and did not systematically generate whole‐genome data, thereby limiting regional phylogenetic and comparative analyses.

In Côte d'Ivoire, sentinel surveillance of acute respiratory infections conducted between 2003 and 2010 in Abidjan and other regions of the country revealed high viral circulation among children with influenza‐like illness, with respiratory pathogens detected in 85% of samples. HPIV accounted for 16% of detections, ranking fourth after respiratory syncytial virus, rhinoviruses, and influenza viruses [8, 9]. Despite documented circulation of respiratory viruses in Côte d'Ivoire, available data remain limited and largely lack genomic characterization [10], reflecting broader gaps in molecular surveillance and restricting comparisons of locally circulating HPIV strains with regional and global evolutionary patterns [10].

Methodologically, conventional unbiased metagenomic sequencing presents important limitations when applied to respiratory samples with low viral loads, a frequent scenario in tropical settings and routine surveillance systems [11]. Hybrid‐capture enrichment strategies provide a robust alternative by increasing the proportion of viral reads, improving whole‐genome recovery, and reducing biases associated with targeted amplicon‐based approaches. Recent studies have demonstrated the effectiveness of such strategies for respiratory virus genomic surveillance, particularly when applied through broad and standardized panels [12, 13, 14]. In this context, hybrid‐capture technology using a respiratory virus enrichment panel represents a promising approach to strengthen HPIV genomic surveillance in resource‐limited settings [15].

The present study aimed to advance genomic surveillance of HPIV in Côte d'Ivoire by evaluating the performance of Twist hybrid‐capture sequencing on routinely collected respiratory samples. Specifically, we assessed the ability of this approach to generate high‐quality genomes across a range of viral loads, characterize the genetic diversity and phylogenetic structure of circulating HPIV strains, and evaluate the added value of hybrid capture for integrated respiratory virus surveillance in a public health context.

## 2 Materials and Methods

### 2.1 Study Design

This study was a cross‐sectional descriptive study conducted at the Respiratory Virus Unit of the Institut Pasteur de Côte d'Ivoire, which serves as the World Health Organization (WHO) National Influenza Centre (NIC) for influenza and other respiratory viruses. The study involved the analysis of epidemiological data and biological specimens collected through the national influenza sentinel surveillance network. The surveillance network was established in accordance with WHO recommendations outlined in the *Protocol for National Influenza Sentinel Surveillance* and comprises 10 sentinel health facilities distributed across different health districts of the country, covering an estimated population of approximately two million inhabitants. These sentinel sites are located across multiple regions of Côte d'Ivoire (Figure 1). The data included in this study were collected over a three‐year period, from January 2022 to December 2024.

**FIGURE 1:**
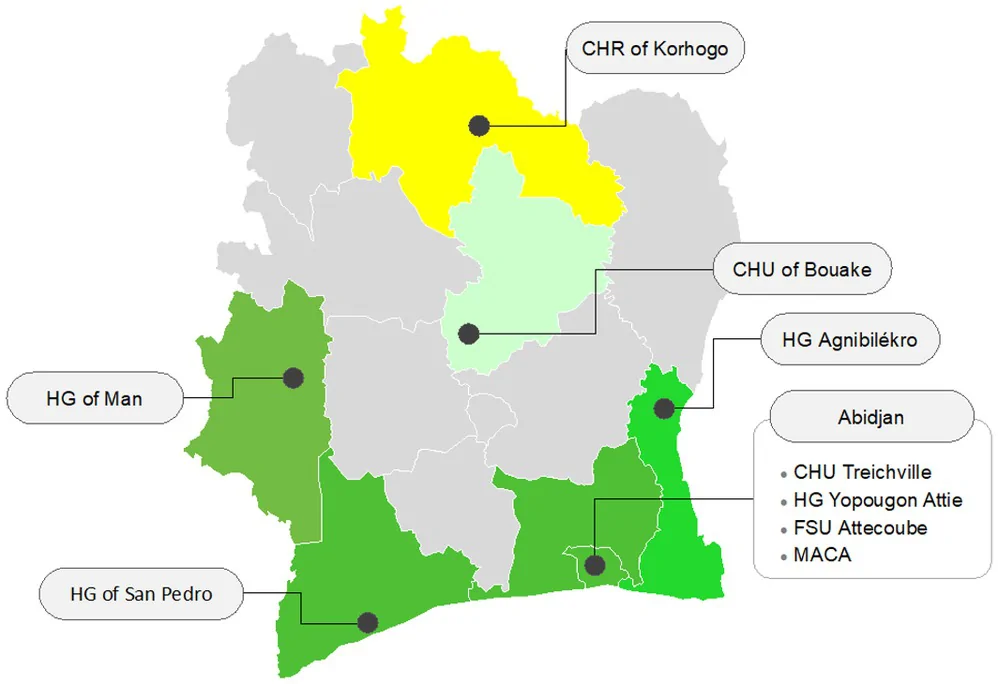
Location of influenza sentinel sites in Côte d'Ivoire. Map showing the geographic distribution of sentinel sites participating in the national influenza surveillance network. Abbreviations: CHR, Centre Hospitalier Régional (Regional Hospital Center); CHU, Centre Hospitalier Universitaire (University Teaching Hospital); FSU, Formation Sanitaire Urbaine (Urban Health Facility); HG, Hôpital Général (General Hospital); MACA, Maison d'Arrêt et de Correction d'Abidjan (Abidjan Prison and Correctional Facility).

### 2.2 Study Population

The study population consisted of patients recruited on a weekly basis at each sentinel site, in accordance with the World Health Organization (WHO) case definitions for influenza‐like illness (ILI) and severe acute respiratory infection (SARI). An ILI case was defined as an acute respiratory illness with measured fever (temperature ≥ 38°C) and cough, with symptom onset within the previous 10 days. A SARI case was defined as an acute respiratory illness with a history of fever or measured fever (≥ 38°C), accompanied by cough and/or shortness of breath, with symptom onset within the previous 10 days, and requiring hospitalization.

### 2.3 Sample Collection

A total of 13,530 nasopharyngeal swabs were collected using the Copan Universal Transport Medium (UTM‐RT) system. The samples were taken from outpatients or hospitalized patients, in accordance with sentinel surveillance procedures. After collection, the samples were immediately stored in a cooler equipped with cold packs or stored at +4°C and then transported as quickly as possible to the reference laboratory at the Institut Pasteur de Côte d'Ivoire for analysis. For each patient included, a standardized epidemiological form was completed, collecting demographic data, medical history, signs of clinical severity, and risk factors associated with respiratory infections. All samples and associated data were collected as part of the national influenza sentinel surveillance system.

### 2.4 RNA Extraction

Viral RNA was extracted from 200 μL of nasopharyngeal swab specimens collected in viral transport medium (VTM). Extraction was performed using the QIAamp Viral RNA Mini Kit (QIAGEN, Hilden, Germany) according to the manufacturer's instructions, with RNA eluted in a final volume of 80 μL of AVE buffer. Nuclease‐free water was included in each extraction batch as a negative control to monitor for potential contamination. Two aliquots were prepared from each extracted RNA sample: One was immediately used for RT‐PCR testing, and the second was stored at −80°C for subsequent analyses.

### 2.5 Detection of HPIV in Nasopharyngeal Samples by RT‐qPCR

Extracted RNA was tested by real‐time reverse transcription polymerase chain reaction (RT‐qPCR) targeting the hemagglutinin‐neuraminidase (HN) gene of HPIV1, HPIV2, and HPIV3. Primers and probes were custom‐synthesized by TIB MOLBIOL Syntheselabor GmbH (Berlin, Germany) and designed against the GenBank reference sequences HPIV1 (U70948), HPIV2 (D00865), and HPIV3 (EU814626). RT‐qPCR reactions were performed in a final volume of 25 μL using reagents from Invitrogen (Thermo Fisher Scientific, USA), according to the manufacturer's instructions. HPIV4 was not targeted by this assay and was therefore outside the scope of the present analysis. RT‐qPCR–positive samples collected during the study period were included in the descriptive analysis, and a subset was selected for whole‐genome sequencing based on cycle threshold (Ct) values.

### 2.6 Next‐Generation Sequencing and Hybrid‐Capture Enrichment

Respiratory specimens from confirmed acute human parainfluenza virus (HPIV) infections were selected for genomic sequencing using a hybrid‐capture workflow for respiratory viruses. Extracted viral RNA was reverse‐transcribed into complementary DNA (cDNA) using the SuperScript IV Reverse Transcriptase kit (Invitrogen, Thermo Fisher Scientific, USA), according to the manufacturer's instructions.

Sequencing libraries were then prepared using the Library Preparation EF 2.0 with Enzymatic Fragmentation and Twist Universal Adapter System protocol (Twist Bioscience). This workflow included enzymatic fragmentation of cDNA, end‐repair, 3′ adenylation (dA‐tailing), ligation of Twist universal adapters, and library amplification. Library quality and concentration were assessed prior to target enrichment, and DNA was quantified using a Qubit fluorometer (Thermo Fisher Scientific, USA), in accordance with the manufacturer's instructions.

Target enrichment was performed by solution‐based hybrid capture using the Twist Respiratory Virus Research (RVR) Target Enrichment Panel (Twist Bioscience, San Francisco, CA, USA), following the manufacturer's recommendations. This panel consists of biotinylated probes designed to cover the complete genomes of a broad range of respiratory viruses, including human parainfluenza viruses. Indexed libraries were pooled, hybridized with capture probes, recovered using streptavidin‐coated magnetic beads, and subjected to post‐capture amplification to enrich viral sequences.

The enriched libraries were sequenced on an Illumina iSeq 100 platform (Illumina, USA) according to the manufacturer's protocols, generating genomic data for downstream genetic and phylogenetic analyses of circulating HPIV strains.

### 2.7 Bioinformatic and Phylogenetic Analysis

Raw sequencing data were generated in FASTQ format, and genome assembly was performed using the CZ‐ID platform (Chan Zuckerberg ID), an open‐source metagenomic analysis tool available at http://czid.org [16]. Default filtering parameters were applied for read quality control, taxonomic assignment, and consensus sequence generation. A minimum depth of coverage of 10× was used as the threshold for base calling. All consensus sequences generated in this study were compared against reference sequences using the NCBI BLASTn web interface to confirm viral identity and subtype classification. Multiple sequence alignments were performed using reference datasets retrieved from public databases. Phylogenetic trees were reconstructed using the maximum likelihood (ML) method implemented in IQ‐TREE [17]. The best‐fitting nucleotide substitution model was selected based on model testing. Node support was assessed using 1000 bootstrap replicates to evaluate the robustness of the inferred phylogenetic relationships.

### 2.8 Statistical Analysis

Sociodemographic and clinical data, including variables such as age, sex, and geographic region, were recorded using Epi Info software (version 7, Centers for Disease Control and Prevention, USA). The dataset was subsequently exported to Microsoft Excel for data management and preliminary processing. Associations between epidemiological and clinical variables and the distribution of HPIV cases were analyzed using R software (version 4.0.2, 64‐bit). Statistical significance was defined as a two‐sided *p* value ≤ 0.05. To explore temporal patterns of HPIV circulation, detections were summarized by month and according to the four local climatic seasons: the major dry season (December–March), major rainy season (April–July), short dry season (August–September), and short rainy season (October–November). Differences in overall HPIV positivity across seasons were assessed using the chi‐square test, with a two‐sided *p* value < 0.05 considered statistically significant.

## 3 Results

### 3.1 Demographic and Clinical Characteristics of Human Parainfluenza Virus Infections

Among the 13,530 respiratory specimens tested, a total of 268 human parainfluenza virus (HPIV) infections were detected. These were distributed as follows: HPIV1 (*n* = 89; 33.2%), HPIV2 (*n* = 64; 23.9%), and HPIV3 (*n* = 115; 42.9%), with HPIV3 being the predominant type. A male predominance was observed, with 164 cases (61.2%) compared to 104 female cases (38.8%). This trend was consistent across all viral types, particularly for HPIV2 and HPIV3. Most cases occurred in children aged 0–2 years (*n* = 151; 56.3%), followed by those aged 3–5 years (*n* = 33; 12.3%). The age distribution differed significantly between viral types (*p* = 0.02), with HPIV3 being particularly frequent among children under 2 years of age (*n* = 83). Other age groups each accounted for less than 12% of total cases. Regarding clinical severity, 77 cases (28.7%) were classified as severe acute respiratory infection (SARI), while 191 cases (71.3%) were classified as influenza‐like illness (ILI), with no statistically significant difference observed between viral types (*p* = 0.10) (Table 1).

**TABLE 1:** Demographic, clinical, and virological characteristics of human parainfluenza virus infection cases (*n* = 268).

Characteristic	HPIV1 *n* = 89 (33.20%)	HPIV2 *n* = 64 (23.88%)	HPIV3 *n* = 115 (42.91%)	*p*	Total HPIV *n* = 268
Gender, *N* (%)					
Female	38	16	50		104
Male	51	48	65		164
Age, *N* (%)					
0–2 ans	46	22	83		151
3–5 ans	14	12	7	0.02	33
6–15 ans	5	5	2		12
16–25 ans	3	5	4		12
26–45 ans	10	13	7		30
46–65 ans	8	6	6		20
> 66 ans	3	1	6		10
Severity					
SARI	32	24	21	0.10	77
ILI	57	40	94		191

### 3.2 Temporal and Seasonal Distribution of HPIV Infections

The monthly distribution of HPIV‐positive cases showed marked temporal variation during the 2022–2024 surveillance period (Figure S1). HPIV3 predominated during April–May 2022 and again during the first months of 2024, whereas HPIV2 circulation was concentrated mainly in 2023, with a pronounced peak in February. HPIV1 showed increased detections during the second half of 2022 and again toward the end of 2024. Overall, these patterns indicate subtype‐specific temporal fluctuations rather than a uniform annual circulation pattern across the three HPIV types.

When detections were grouped according to the four local climatic seasons, overall HPIV positivity differed significantly across seasons (χ^2^ = 49.45, df = 3, *p* < 0.001). Positivity was highest during the major dry season (122/4087; 2.99%), followed by the major rainy season (101/4827; 2.09%) and the short rainy season (34/2539; 1.34%), and was lowest during the short dry season (11/2077; 0.53%) (Table S1).

### 3.3 Sequence Identity Analysis

The Ct values of the 268 RT‐qPCR‐positive samples ranged from 16 to 38, reflecting substantial variability in viral load within the study population (Table 2). Based on these Ct values, a subset of 49 samples was selected for whole‐genome sequencing. Among these, 29 near‐complete HPIV genomes were successfully recovered from clinical specimens, including 9 HPIV1, 10 HPIV2, and 10 HPIV3 sequences. For HPIV1, nucleotide identity among the nine sequences ranged from 96.2% to 100%. When compared to the prototype strain HPIV1/USA/1964 (GenBank accession AF457102), nucleotide identity ranged from 95.1% to 95.6%. For HPIV2, nucleotide identity among the 10 sequences ranged from 93.1% to 99.5%. Compared to the prototype strain HPIV2/USA/GREER/1955 (AF533012), nucleotide identity ranged from 94.6% to 96.9%. For HPIV3, nucleotide identity among the 10 sequences ranged from 97.3% to 100%. When compared to the prototype strain HPIV3/USA/2006 (KF530247), nucleotide identity ranged from 90% to 95%.

**TABLE 2:** Distribution of RT‐qPCR positive samples according to threshold (Ct) values.

Ct value classes	Number of samples	Percentage (%)
Ct ≤ 25	94	35.1
25 < Ct ≤ 30	85	31.7
30 < Ct ≤ 35	71	26.5
Ct > 35	18	6.7
Total	268	100

### 3.4 Performance of Hybrid‐Capture Sequencing According to Viral Load

Stratified analysis by Ct classes showed that samples with Ct ≤ 25 achieved a mean genome coverage of 97.3%, with a median of 99.3%. Genome coverage declined for samples with Ct values between 25 and 30 (mean coverage: 88.8%). In contrast, samples with Ct ≥ 30 exhibited a marked decline in coverage, with a median coverage of 0% in both the 30 < Ct ≤ 35 and Ct > 35 categories, highlighting the strong influence of viral load on sequencing performance (Table 3). A strong negative correlation was observed between Ct value and genome coverage (*r* = −0.91), indicating a substantial reduction in genome recovery as Ct values increased. Samples with low Ct values consistently yielded near‐complete genomes, whereas higher Ct values were associated with markedly reduced or absent genomic coverage (Figure 2).

**TABLE 3:** Genomic coverage of HPIV sequences according to threshold (Ct) values.

Ct value classes	Number of samples	Average coverage (%)	Median coverage (%)
Ct ≤ 25	17	97.3	99.3
25 < Ct ≤ 30	9	88.8	91.2
30 < Ct ≤ 35	8	14.8	0
Ct > 35	15	0	0

**FIGURE 2:**
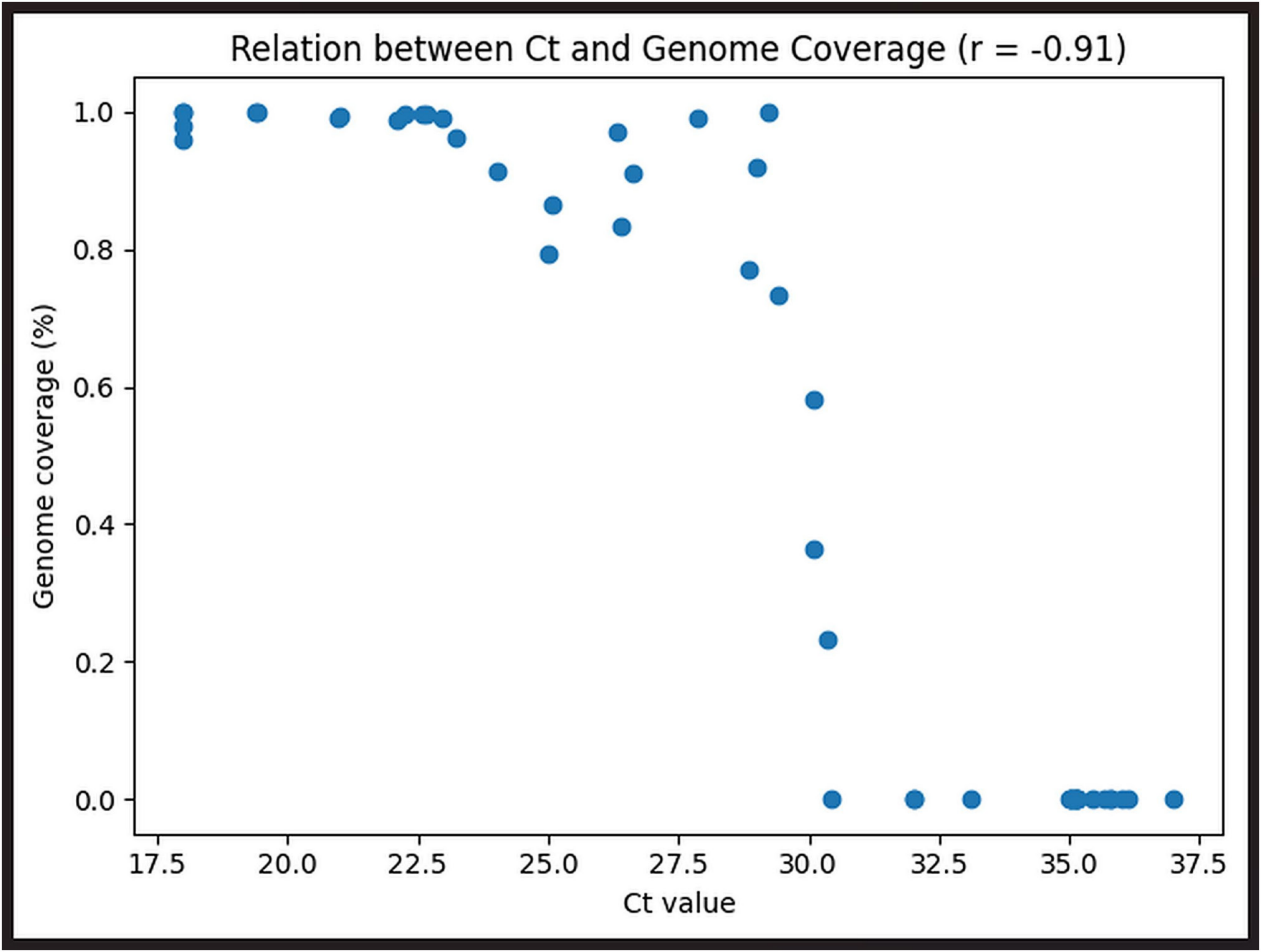
Genome coverage according to cycle threshold (Ct) values among sequenced HPIV‐positive samples. Each dot represents one sample. Genome coverage declined as Ct values increased, with a strong negative correlation between Ct and coverage (*r* = −0.91). Samples with low Ct values generally achieved near‐complete genome recovery, whereas samples with higher Ct values showed limited or no coverage.

### 3.5 Viral Co‐Detections Associated With Human Parainfluenza Viruses

During the study period, 49 HPIV‐positive samples were sequenced and analyzed, revealing a total of 25 viral co‐detections. Among these, five samples (10.2%) exhibited multiple co‐infections involving more than two distinct respiratory viruses, highlighting the complexity of viral respiratory infections in the study population. Among viruses co‐detected with HPIV, human adenovirus type B was the most frequently identified pathogen, accounting for 20% of co‐detections (*n* = 5). Influenza A and Influenza B viruses were detected in three and two samples, respectively, collectively representing 20% of co‐infections. Respiratory syncytial virus (RSV), rhinoviruses, and betacoronaviruses were each identified in two samples (8% each). Additional less frequent pathogens included enteroviruses, bocavirus, *Haemophilus* spp., human coronavirus HKU1, and notably, one case of co‐infection with measles virus genotype B3. Analysis of specific associations between HPIV types and other respiratory viruses revealed distinct co‐detection patterns. HPIV1 showed a relatively heterogeneous distribution, with seven different viruses each detected once in co‐infected samples. HPIV2 was predominantly associated with adenovirus B, identified in three of the five HPIV2 co‐detections (60%). HPIV3, being the most prevalent type, exhibited the broadest spectrum of co‐infections, involving 11 different respiratory viruses, with a slight predominance of influenza A viruses and rhinoviruses (two cases each). Cases of multiple co‐infections warrant particular attention. One sample revealed a triple infection involving HPIV3 and HPIV1 in association with influenza B virus. Additionally, four samples exhibited co‐infections with at least two other respiratory viruses alongside HPIV, suggesting either increased host susceptibility to multiple viral infections or epidemiological conditions favoring simultaneous circulation of several respiratory pathogens (Figure 3).

**FIGURE 3:**
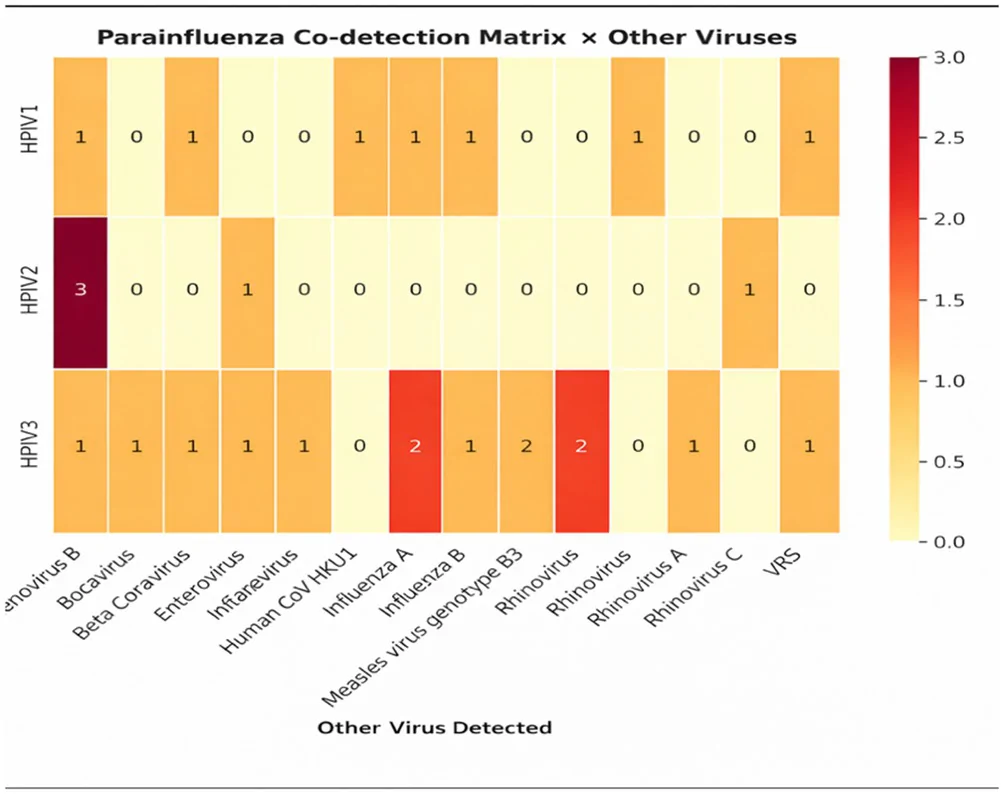
Profile of viral co‐detections associated with parainfluenza virus (HPIV) infections. Heatmap illustrating the distribution and frequency of viral co‐detections among sequenced HPIV‐positive samples according to HPIV type. Each cell shows the number of co‐detections observed for a given virus–HPIV combination, with darker colors indicating higher frequencies. HPIV3 exhibited the most diverse co‐detection profile, while HPIV2 was most frequently associated with adenovirus B.

### 3.6 Phylogenetic Analysis of Human Parainfluenza Virus Strains

Sequencing data generated in this study enabled the reconstruction of phylogenetic trees illustrating the evolutionary relationships among the detected viral strains.

#### 3.6.1 Phylogenetic Characterization of HPIV1 Strains

The accession numbers corresponding to all Ivorian HPIV1, HPIV2, and HPIV3 sequences included in the phylogenetic analyses are provided in Table S2. Phylogenetic analysis of HPIV1 strains detected during 2022–2023 showed limited genetic diversity. The maximum‐likelihood tree identified four major clusters (A–D) among reference sequences spanning 1964–2024. The Ivorian HPIV1 strains were distributed across two groups, with GR3851 and GR3858 clustering within group D and the remaining strains within cluster C. This pattern indicates the co‐circulation of at least two closely related lineages during the study period. The group C Ivorian strains clustered with recent reference sequences from several geographic regions, including Europe, Asia, and North America, whereas GR3851 and GR3858 formed part of a distinct group containing recent African and Asian reference sequences. Overall, the relatively short branch lengths are consistent with limited genetic divergence among the circulating HPIV1 strains (Figure 4).

**FIGURE 4:**
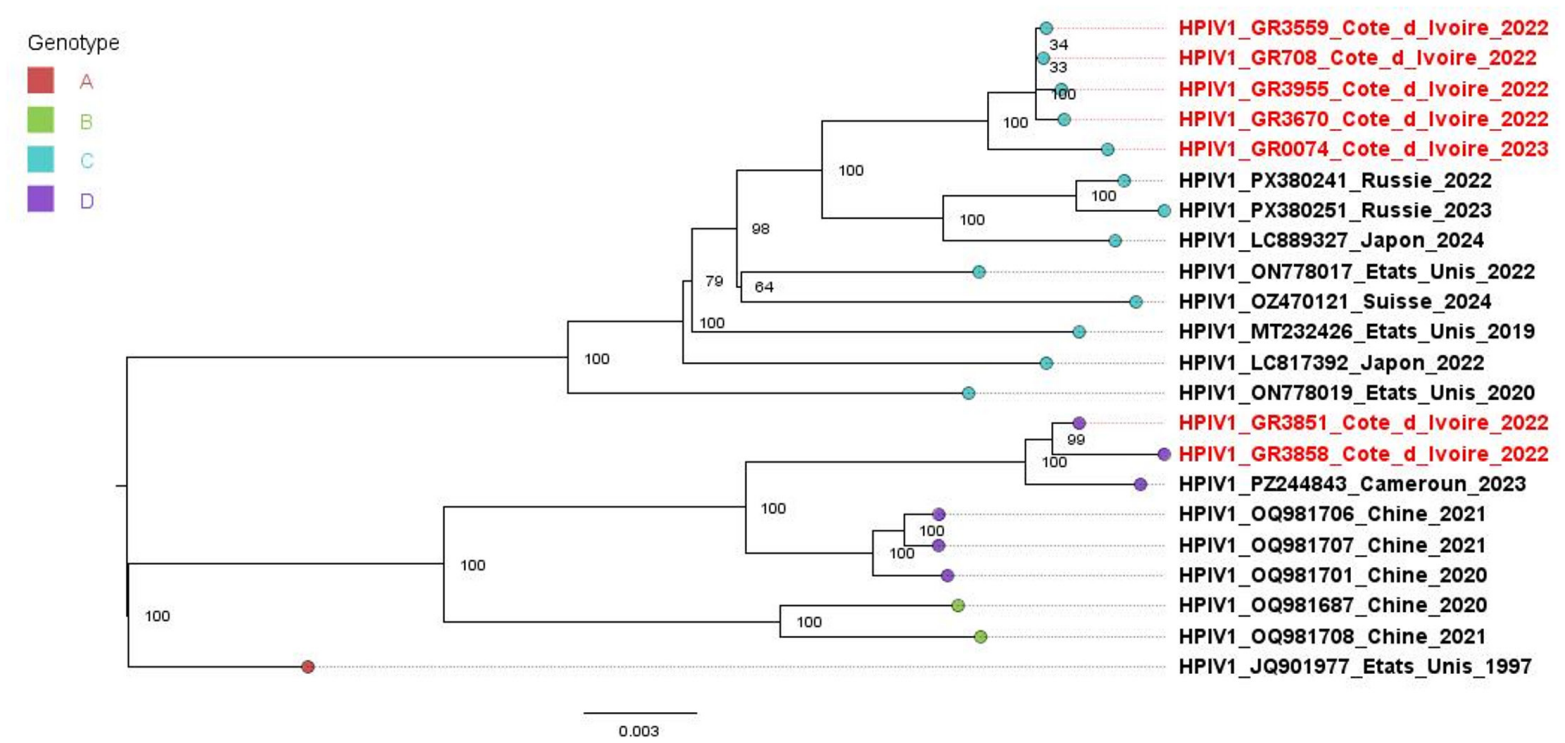
Maximum‐likelihood phylogenetic tree of near‐complete HPIV1 genomes, including Ivorian strains and international reference sequences. The tree was reconstructed from near‐complete genomes of human parainfluenza virus type 1 (HPIV1) using the maximum‐likelihood method. Sequences generated in this study are shown in red, whereas reference sequences from public databases are shown in black. Four major genetic clusters (A–D) are indicated by color coding. Branch lengths represent the number of nucleotide substitutions per site, and the scale bar corresponds to 0.003 substitutions per site. Bootstrap support values are shown at major nodes when they exceeded the selected threshold. The Ivorian sequences are distributed across two clusters, with GR3851 and GR3858 grouping within cluster D and the remaining sequences within cluster C.

#### 3.6.2 Phylogenetic Characterization of HPIV2 Strains

Phylogenetic analysis of HPIV2 strains identified during 2023–2024 revealed greater genetic diversity than that observed for HPIV1. The maximum‐likelihood phylogenetic tree identified four major groups or subgroups (G1a, G1e, G3, and G4) among reference sequences spanning 1955–2024. The Ivorian HPIV2 strains were distributed across three of these groups. Four strains collected in 2023 (GR2865, GR0468, GR0379, and GR0378) clustered within G3, together with recent reference sequences including strains from Russia. GR0644 grouped within G1e, whereas GR0364, GR0723, and GR0082 grouped within G1a alongside recent reference sequences from several geographic regions. This distribution indicates the co‐circulation of multiple genetically distinct HPIV2 lineages in Côte d'Ivoire during the study period (Figure 5).

**FIGURE 5:**
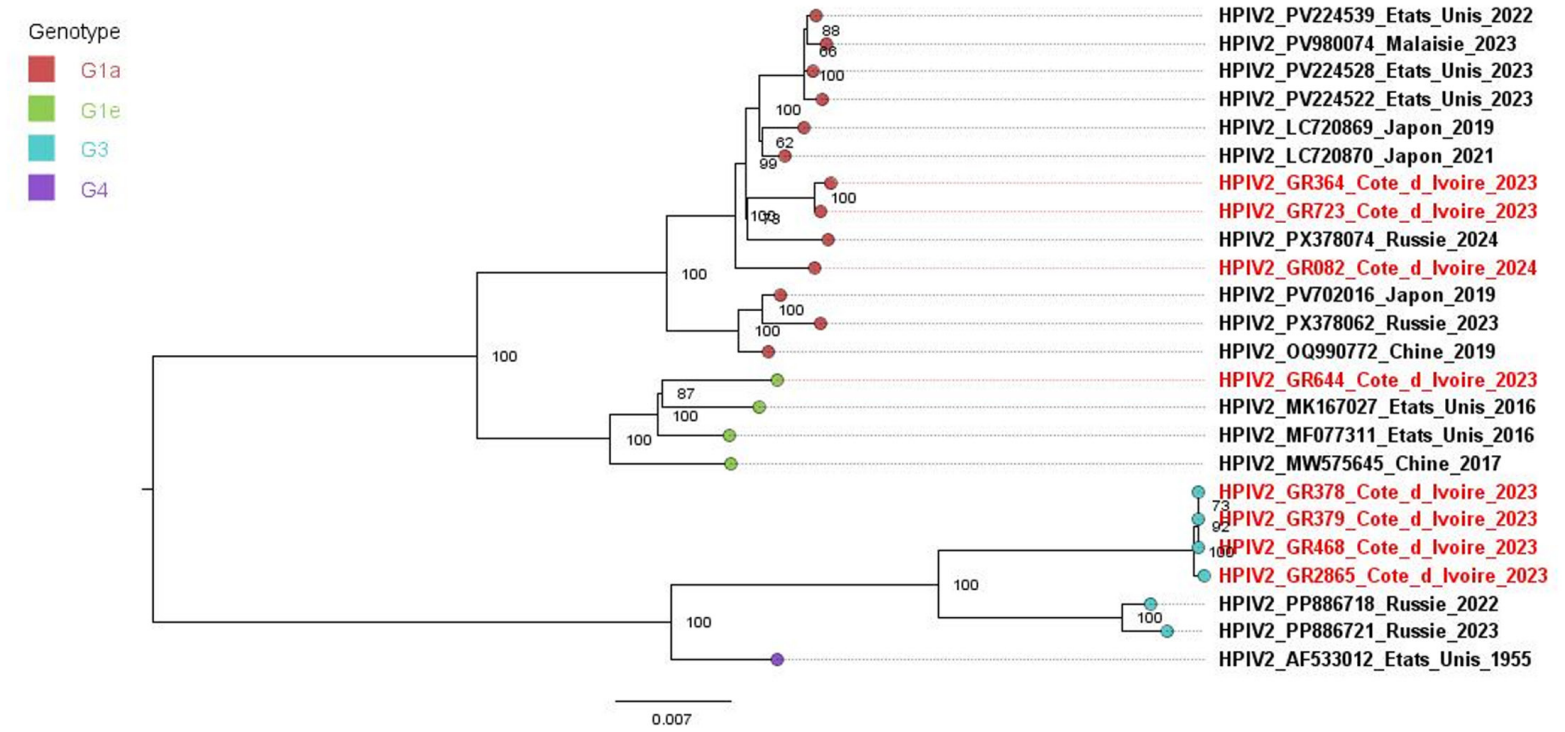
Maximum‐likelihood phylogenetic tree of near‐complete HPIV2 genomes, including Ivorian strains and international reference sequences. The tree was reconstructed from near‐complete genomes of human parainfluenza virus type 2 (HPIV2) using the maximum‐likelihood method. Sequences generated in this study are shown in red, whereas reference sequences are shown in black. Major genetic groups and subgroups (G1a, G1e, G3, and G4) are indicated by color coding. Branch lengths represent the number of nucleotide substitutions per site, and the scale bar corresponds to 0.007 substitutions per site. The Ivorian sequences are distributed across G1a, G1e, and G3, consistent with the co‐circulation of multiple HPIV2 lineages in Côte d'Ivoire during 2023–2024.

#### 3.6.3 Phylogenetic Characterization of HPIV3 Strains

Phylogenetic analysis of HPIV3 strains identified during 2022–2024 revealed the greatest phylogenetic heterogeneity among the three HPIV types analyzed. The maximum‐likelihood tree included several genetic groups and subgroups (A, C1c, C3a, C3b, C3e, C3f, C5, and D) among reference sequences spanning 1957–2024. The Ivorian HPIV3 strains were distributed across four distinct groups. GR2232 grouped within C3a, while GR0979, GR0996, and GR1369 clustered within C3b. GR1361 grouped within C3e, whereas GR2141 and GR3822 clustered within group D. The distribution of Ivorian strains across several phylogenetically distant groups despite overlapping sampling periods demonstrates the co‐circulation of genetically heterogeneous HPIV3 lineages in Côte d'Ivoire during the study period (Figure 6).

**FIGURE 6:**
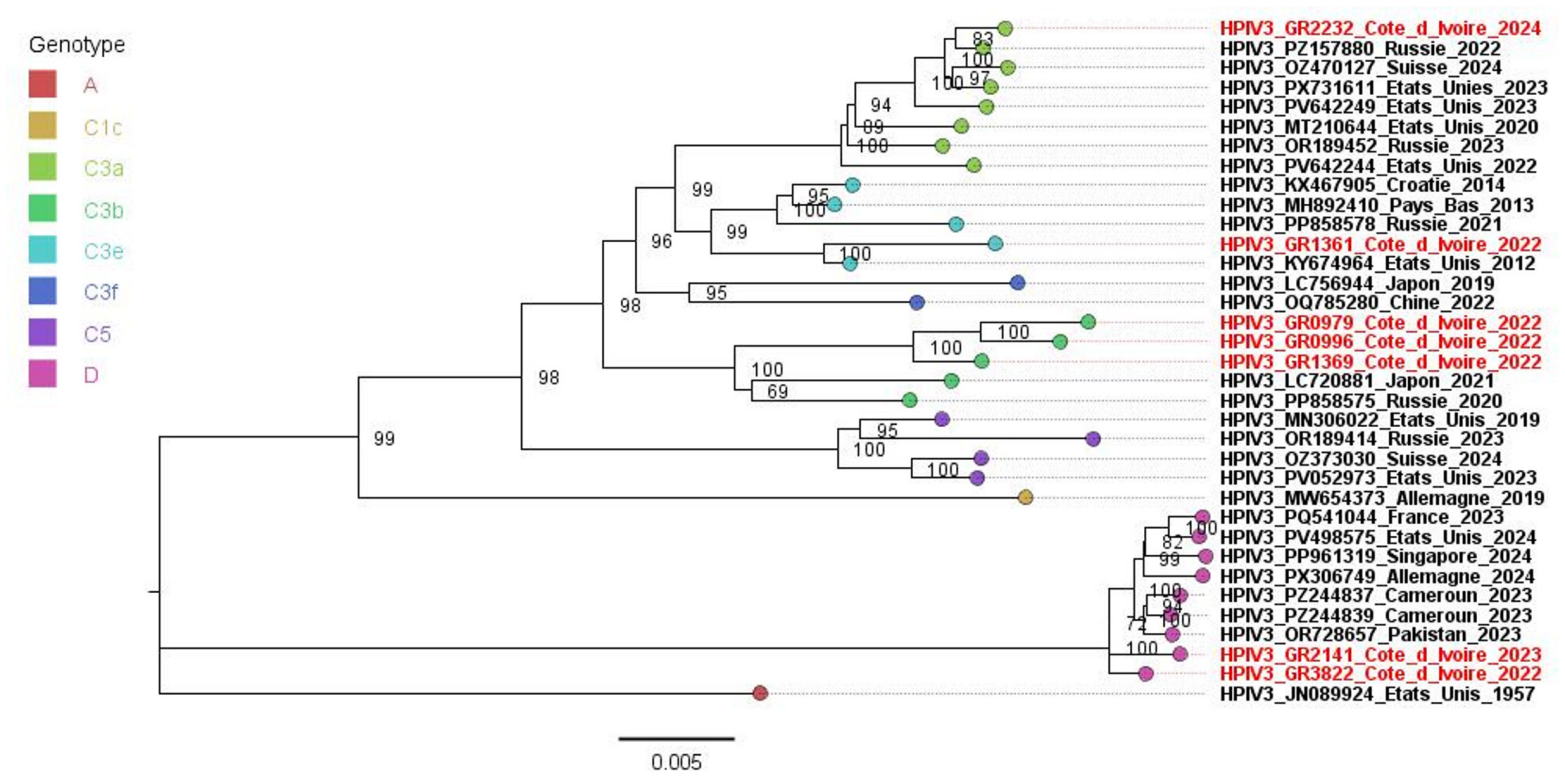
Maximum‐likelihood phylogenetic tree of near‐complete HPIV3 sequences, including Ivorian strains and international reference sequences. The tree was reconstructed from near‐complete genomes of human parainfluenza virus type 3 (HPIV3) using the maximum‐likelihood method. Sequences generated in this study are shown in red, whereas reference sequences retrieved from public databases are shown in black. Major genetic groups and subgroups (A, C1c, C3a, C3b, C3e, C3f, C5, and D) are indicated by color coding. Branch lengths represent the number of nucleotide substitutions per site, and the scale bar corresponds to 0.005 substitutions per site. Bootstrap support values are shown at the main nodes when they exceeded the selected threshold. The Ivorian sequences are distributed across C3a, C3b, C3e, and D, illustrating the co‐circulation of genetically distinct HPIV3 lineages.

## 4 Discussion

The findings of this study demonstrate a strong negative relationship between Ct value and genomic coverage, confirming that viral load is a major determinant of sequencing success, as widely reported for next‐generation sequencing approaches applied to respiratory viruses [18, 19]. Lower Ct values were associated with improved genome recovery, whereas increasing Ct values led to a progressive decline in sequencing depth and genome completeness. Importantly, the recovery of usable genomes from samples with Ct values approaching 30 highlights a key advantage of hybrid‐capture sequencing over conventional amplicon‐based methods. Unlike targeted amplification strategies, which are often limited by RNA degradation or primer mismatches, hybrid capture enriches viral fragments across the genome and therefore improves the likelihood of successful sequencing from specimens with lower viral loads [12].

The variability in genomic coverage observed among samples with similar Ct values indicates that Ct alone is not an absolute predictor of sequencing performance. Pre‐analytical factors such as RNA integrity, sample transport and storage conditions, as well as library preparation and capture efficiency, may substantially influence sequencing outcome [20]. This observation is particularly relevant in routine surveillance settings, where sample handling conditions are often heterogeneous. Overall, our results indicate that Twist hybrid capture extends the practical sequencing window beyond traditional next‐generation sequencing thresholds, improves genomic representativeness, reduces selection bias related to viral load, and strengthens the detection of circulating genetic diversity, in line with recommendations for integrated genomic surveillance systems [21, 22].

This study also revealed a notable frequency of viral co‐detections involving HPIV, consistent with the growing body of literature emphasizing the epidemiological relevance of polymicrobial respiratory infections. The predominance of HPIV3 in our cohort aligns with international surveillance data identifying HPIV3 as the most frequently detected serotype [1]. Early acquisition of HPIV3 during childhood may partly explain its frequent involvement in co‐infections, given its sustained circulation in pediatric populations [23, 24]. Adenovirus type B was the most frequently co‐detected pathogen, in agreement with recent reports describing frequent HPIV–adenovirus co‐infections [25, 26]. Such co‐detections may be clinically relevant, as they have been associated with increased disease severity, thereby underscoring the importance of comprehensive multiplex diagnostic approaches in pediatric respiratory infections.

Co‐detections with influenza A and B viruses are also noteworthy. Experimental evidence suggests that influenza virus co‐infection may enhance the replication of certain HPIV types, indicating possible synergistic viral interactions [27]. In addition, viral co‐infections have been associated with higher rates of intensive care admission and increased mortality in community‐acquired pneumonia. The detection of rhinovirus in several cases further supports its recognized role in polymicrobial respiratory infections and, as a rapidly replicating virus, rhinovirus may influence co‐infection dynamics through mechanisms of viral interference or competitive replication [25]. The identification of triple infections further illustrates the complexity of respiratory infections in the study population and may reflect immune immaturity, host susceptibility, or intense simultaneous circulation of multiple pathogens.

Importantly, the observed co‐detection profile likely reflects, at least in part, the background circulation of other respiratory pathogens during the same periods. Co‐detections may therefore be more frequently observed when HPIV and other respiratory viruses are simultaneously circulating at higher levels in the population. Because the present analysis did not include time‐matched circulation data for all co‐detected pathogens across the full surveillance population, we cannot distinguish co‐detections driven primarily by overlapping circulation from those potentially reflecting specific biological interactions. The observed co‐detection patterns should therefore be interpreted within the broader epidemiological context of respiratory virus co‐circulation.

The co‐detection of measles virus genotype B3 with HPIV3 represents an unusual observation. Although both viruses belong to the family *Paramyxoviridae*, documented co‐circulation remains uncommon. This finding highlights the value of integrated virological surveillance and reinforces the importance of maintaining high measles vaccination coverage, particularly in settings where multiple respiratory viruses co‐circulate. In addition, the detection of *Haemophilus influenzae* emphasizes the need to consider bacterial co‐infections when interpreting respiratory disease severity.

The temporal analysis further revealed subtype‐specific fluctuations in HPIV circulation across the three‐year surveillance period. Rather than a single consistent annual peak shared by all three types, HPIV1, HPIV2, and HPIV3 showed different periods of increased activity, while overall HPIV positivity also varied significantly across local climatic seasons. These observations suggest that HPIV circulation in Côte d'Ivoire may be influenced by seasonal and climatic factors, although longer‐term surveillance will be required to determine whether the observed fluctuations represent stable and recurrent subtype‐specific seasonal patterns, as reported in both temperate and tropical settings [28, 29].

From an evolutionary perspective, comparative phylogenetic analysis revealed clear differences among the three serotypes. HPIV1 showed limited genetic diversity, consistent with the co‐circulation of a small number of closely related lineages and sustained local transmission. This pattern aligns with earlier genomic analyses indicating that HPIV1 evolution is often characterized by a restricted number of clades, with evidence of local persistence and, in some settings, geographic structuring rather than rapid, extensive diversification [30, 31]. In contrast, HPIV2 displayed intermediate diversity, with co‐circulating related lineages suggesting either multiple introductions or gradual local diversification. This pattern is consistent with recent whole‐genome analyses showing that HPIV2 circulation may involve several co‐circulating genotypes and subgenotypes with changing temporal distributions, rather than a single homogeneous lineage [32]. HPIV3 exhibited the greatest complexity, with several highly divergent lineages showing phylogenetic affinities with strains from different geographic regions, confirming its status as the most genetically diverse and epidemiologically dynamic serotype identified in this study. This pattern is in line with recent genomic analyses showing that HPIV3 comprises multiple major clusters and subclusters with substantially greater interclade divergence than HPIV1 and HPIV2 and undergoes continuous evolution partly shaped by hypervariable genomic regions that contribute to lineage diversification [33, 34].

Taken together, these findings indicate distinct circulation dynamics among HPIV serotypes in Côte d'Ivoire. In particular, the co‐circulation of divergent HPIV3 lineages during overlapping sampling periods may reflect repeated introductions and/or sustained regional circulation of multiple variants. Such diversity may influence viral persistence, transmission dynamics, and immune escape, reinforcing the need for sustained molecular surveillance.

These phylogenetic differences are also relevant for vaccine development. Although no licensed vaccine against HPIV is currently available, several candidates targeting the HN and F glycoproteins are under development, notably live‐attenuated intranasal vaccines for HPIV2 and a diverse pipeline of prophylactic approaches for HPIV3, including vector‐based, subunit, mRNA, and antibody‐based strategies [35, 36]. The relative homogeneity observed for HPIV1 may theoretically favor the effectiveness of a vaccine based on a representative dominant lineage. By contrast, the higher genetic diversity observed for HPIV3 may reduce the breadth of protection provided by a monovalent vaccine strategy, particularly if amino acid variation affects antigenically important regions of the HN and F proteins. Given that these glycoproteins remain central targets for HPIV vaccine development, including in recent HPIV3 candidate studies, continued diversification in these regions could argue for broader or multivalent vaccine designs [37, 38]. This situation parallels challenges observed with other antigenically evolving respiratory viruses, where multivalent formulations or vaccines targeting conserved regions may be preferable [3, 39].

In this context, strengthening genomic surveillance in sub‐Saharan Africa is essential to document local viral diversity, detect emerging variants early, and inform the rational design of future vaccine candidates adapted to regional epidemiological realities. More broadly, our findings support the integration of hybrid‐capture sequencing into respiratory virus surveillance frameworks, particularly in settings where viral loads are variable and genomic data remain scarce. Such approaches may improve understanding of viral ecology, refine transmission analyses, and contribute to more responsive public health strategies grounded in robust local data.

One important limitation of this study is that only a subset of RT‐qPCR‐positive samples was sequenced, which may have reduced the representativeness of the recovered genomes, particularly for samples with higher Ct values. A further limitation is that the molecular screening assay targeted HPIV1, HPIV2, and HPIV3 only. HPIV4 was not included in the assay design; therefore, the present study cannot assess its circulation in Côte d'Ivoire. Although significant temporal and seasonal fluctuations were observed, the three‐year surveillance period limits our ability to determine whether these patterns represent stable long‐term seasonality. In addition, the interpretation of co‐detections remains limited in the absence of detailed clinical severity analyses and time‐matched circulation data for the other respiratory pathogens detected. Consequently, the present study cannot distinguish active biological interactions from co‐detections resulting from overlapping circulation, prolonged shedding, or incidental detection. Nevertheless, this study provides valuable genomic data on HPIV circulation in Côte d'Ivoire and contributes to addressing the major underrepresentation of African HPIV sequences in public databases.

## 5 Conclusion

Hybrid‐capture sequencing was most effective in specimens with low to moderate viral loads, whereas genome recovery declined sharply as Ct values increased. These findings confirm the strong influence of viral load on sequencing success and support the use of hybrid‐capture approaches for respiratory virus genomic surveillance, particularly in settings where sample quality and viral concentration are variable.

This study also highlights the complexity of HPIV‐associated co‐detections, with HPIV3 showing the broadest co‐detection profile and adenovirus type B emerging as the most frequent associated pathogen. Phylogenetic analysis further revealed distinct evolutionary dynamics among HPIV1, HPIV2, and HPIV3, with HPIV3 showing the greatest diversity and epidemiological complexity. In a context where African genomic data remain underrepresented, these findings support strengthened sequencing capacity and sustained regional genomic surveillance as essential components of responsive, evidence‐based public health action.

## Author Contributions

**Olivia Cariolh Koumba‐Koumba:** writing – original draft. **Yakoura Karidja Ouattara:** data curation, formal analysis, software, writing – review and editing. **Herve A. Kadjo:** conceptualization, writing – review and editing, methodology. **Mouhamed Kane:** data curation, formal analysis, software. **Maxime Diané Kouao:** investigation. **Helene Kouassi:** investigation. **Ange‐Michele Mbra:** data curation. **Alida Mouliom:** data curation. **Ndeye Awa Ndiaye:** software, formal analysis. **Ndongo Dia:** supervision. **Daouda Coulibaly:** investigation, resources. **Edgard Adjogoua:** validation. **Therese K. Agbessi:** validation. **Moussa Moise Diagne:** formal analysis, software, resources, writing – review and editing, supervision.

## Funding

This work was funded in part by the Africa Pathogen Genomics Initiative (Africa PGI), supported by the Bill & Melinda Gates Foundation (grants INV‐018278, INV‐036413, and INV‐036379), by a direct Bill & Melinda Gates Foundation grant to Institut Pasteur de Dakar (INV‐018978), and the Pasteur Institute of Côte d'Ivoire internal funds. The funders had no role in study design, data collection, analysis, decision to publish, or preparation of the manuscript.

## Ethics Statement

The sentinel surveillance of influenza and other respiratory viral infections is officially endorsed by the Ministry of Health of Côte d'Ivoire. This protocol is exempt from ethical considerations because it falls under public health surveillance. Written informed consent was obtained from all participants, and data were anonymized to ensure confidentiality.

## Conflicts of Interest

The authors declare no conflicts of interest.

## Supporting information


**Table S1:** Seasonal distribution and positivity of human parainfluenza virus infections in Côte d'Ivoire, 2022–2024. Overall HPIV positivity differed significantly across the four climatic seasons (chi‐square test, χ^2^ = 49.45, df = 3, *p* < 0.001).

**Table S2:** Accession numbers for Ivorian HPIV sequences included in the phylogenetic analyses (Figures 4–6).

**Figure S1:** Monthly distribution of HPIV1, HPIV2, and HPIV3 detections in Côte d'Ivoire from January 2022 to December 2024. Monthly numbers of RT‐qPCR‐positive detections are shown for each HPIV type. Local climatic seasons are indicated above the plot: MDS, major dry season (December–March); MRS, major rainy season (April–July); SDS, short dry season (August–September); and SRS, short rainy season (October–November). The figure illustrates marked temporal and subtype‐specific fluctuations in HPIV circulation during the three‐year surveillance period.

## Data Availability

The consensus sequences generated in this study were deposited in GenBank under accession numbers QB076841–QB076862; individual sequence accession numbers are provided in Table S2.
