# Supplementary material for: Advancing Genomic Surveillance of Human Parainfluenza Viruses in Côte d'Ivoire Using Twist Hybrid‐Capture Sequencing
Source: Influenza Other Respir Viruses. 2026 Oct 14;20(10):e70335. doi: 10.1111/irv.70335 (PMC13662739; doi:10.1111/irv.70335)
Supplement: Supplementary file 1 [file IRV-20-e70335-s001.docx]

**Table S1. Seasonal distribution and positivity of human parainfluenza virus infections in Côte d’Ivoire, 2022–2024. Overall HPIV positivity differed significantly across the four climatic seasons (chi-square test, χ² = 49.45, df = 3, p < 0.001).**

| **Season** | **HPIV-positive** | **Samples tested** | **HPIV-negative** | **Positivity (%)** |
| --- | --- | --- | --- | --- |
| Major dry season | 122 | 4,087 | 3,965 | 2.99 |
| Major rainy season | 101 | 4,827 | 4,726 | 2.09 |
| Short dry season | 11 | 2,077 | 2,066 | 0.53 |
| Short rainy season | 34 | 2,539 | 2,505 | 1.34 |
| Total | 268 | 13,530 | 13,262 | 1.98 |

Overall HPIV positivity differed significantly across the four climatic seasons (chi-square test, χ² = 49.45, df = 3, p < 0.001).
