# Supplementary material for: Advancing Genomic Surveillance of Human Parainfluenza Viruses in Côte d'Ivoire Using Twist Hybrid‐Capture Sequencing
Source: Influenza Other Respir Viruses. 2026 Oct 14;20(10):e70335. doi: 10.1111/irv.70335 (PMC13662739; doi:10.1111/irv.70335)
Supplement: Supplementary file 2 [file IRV-20-e70335-s002.docx]

**Supplementary Table S2. Accession numbers for Ivorian HPIV sequences included in the phylogenetic analyses (Figures 4–6).**

| **Virus** | **Sequence label in phylogenetic tree** | **Accession number** |
| --- | --- | --- |
| HPIV1 | HPIV1_GR3559_Cote_d_Ivoire_2022 | QB076843 |
| HPIV1 | HPIV1_GR708_Cote_d_Ivoire_2022 | QB076842 |
| HPIV1 | HPIV1_GR3955_Cote_d_Ivoire_2022 | QB076847 |
| HPIV1 | HPIV1_GR3670_Cote_d_Ivoire_2022 | QB076844 |
| HPIV1 | HPIV1_GR0074_Cote_d_Ivoire_2023 | QB076841 |
| HPIV1 | HPIV1_GR3851_Cote_d_Ivoire_2022 | QB076845 |
| HPIV1 | HPIV1_GR3858_Cote_d_Ivoire_2022 | QB076846 |
| HPIV2 | HPIV2_GR364_Cote_d_Ivoire_2023 | QB076849 |
| HPIV2 | HPIV2_GR723_Cote_d_Ivoire_2023 | QB076854 |
| HPIV2 | HPIV2_GR082_Cote_d_Ivoire_2024 | QB076848 |
| HPIV2 | HPIV2_GR644_Cote_d_Ivoire_2023 | QB076853 |
| HPIV2 | HPIV2_GR378_Cote_d_Ivoire_2023 | QB076850 |
| HPIV2 | HPIV2_GR379_Cote_d_Ivoire_2023 | QB076851 |
| HPIV2 | HPIV2_GR468_Cote_d_Ivoire_2023 | QB076852 |
| HPIV2 | HPIV2_GR2865_Cote_d_Ivoire_2023 | QB076855 |
| HPIV3 | HPIV3_GR2232_Cote_d_Ivoire_2024 | QB076861 |
| HPIV3 | HPIV3_GR1361_Cote_d_Ivoire_2022 | QB076858 |
| HPIV3 | HPIV3_GR0979_Cote_d_Ivoire_2022 | QB076856 |
| HPIV3 | HPIV3_GR0996_Cote_d_Ivoire_2022 | QB076857 |
| HPIV3 | HPIV3_GR1369_Cote_d_Ivoire_2022 | QB076859 |
| HPIV3 | HPIV3_GR2141_Cote_d_Ivoire_2023 | QB076860 |
| HPIV3 | HPIV3_GR3822_Cote_d_Ivoire_2022 | QB076862 |
